# Author Correction: Phosphorus-solubilizing bacteria improve the growth of *Nicotiana benthamiana* on lunar regolith simulant by dissociating insoluble inorganic phosphorus

**DOI:** 10.1038/s42003-026-10421-7

**Published:** 2026-06-16

**Authors:** Yitong Xia, Yu Yuan, Chenxi Li, Zhencai Sun

**Affiliations:** 1 College of Agronomy and Biotechnology, China Agricultural University, Haidian District, Beijing, China; 2 College of Engineering, China Agricultural University, Haidian District, Beijing, China; 3 College of Horticulture, China Agricultural University, Haidian District, Beijing, China

Correction to: *Communications Biology* 10.1038/s42003-023-05391-z, published online 09 November 2023

In the version of this article initially published, there were labeling errors in Fig. 1a, where the sizes of simulants on the plates, now reading top-down as “0.3–0.5 mm; 0.5–0.9 mm; >5 mm” appeared originally as “>5 mm; 0.5–0.9 mm; 0.3–0.5 mm.” The figure and associated legend text have been amended in the HTML and PDF versions of the article.

