# Supplementary material for: Phosphorus-solubilizing bacteria improve the growth of Nicotiana benthamiana on lunar regolith simulant by dissociating insoluble inorganic phosphorus
Source: Commun Biol. 2023 Nov 9;6:1039. doi: 10.1038/s42003-023-05391-z (PMC10636133; doi:10.1038/s42003-023-05391-z)
Supplement: Supplementary file 2 — Supplementary Materials [file 42003_2023_5391_MOESM2_ESM.pdf]

Supplementary Measurement Methods

1. Analysis of the growth curve. Considering the influence of accumulation of colored components in liquid medium on its light absorption properties, the OD_{600} values of the culture liquid samples and their corresponding supernatant after centrifugation were measured on a microplate reader(Multiskan GO, Thermo Fisher Scientific), which were respectively labeled as A_S and A_C. We believe that the difference between A_S and A_C of a sample can be used as a corrected value for microbial growth status in this sample.
For a certain replicate at a certain culture time, its corrected OD_{600} is labeled as α, and the corrected OD_{600} of its corresponding control group is labeled as β, then the growth inhibition rate(GIR) of this replicate at this moment is defined as R, which can be calculated by the following formula:

$$(1)\ R = \left(1 - \frac{\alpha}{\beta}\right) \times 100\%$$

2. The classification of phosphorus forms. In this study, phosphorus was classified into four categories: 1) insoluble phosphorus, which was the main form of phosphorus in the untreated regolith of the lunar surface, and was firmly locked in mineral crystals and unable to be used by plants; 2) dissolved phosphorus, that included phosphorus dissolved in culture medium; 3) microbial biomass phosphorus (MBP), that referred to the phosphorus absorbed by microorganisms, stored in cells and used for life activities; 4) adsorbed phosphorus (AP), that referred to the phosphorus adsorbed on the surface of regolith simulant particles through ligand adsorption or ion exchange adsorption, and could be extracted by $NaHCO_3$ solution. In the more detailed classification, dissolved phosphorus was divided into dissolved inorganic phosphorus (DIP) and dissolved organic phosphorus (DOP), and microbial phosphorus was divided into suspended microbial biomass phosphorus (SMBP) and attached microbial biomass phosphorus (AMBP) according to whether the PSB cells were attached on the simulant particles.
The detailed measurement methods of different forms of phosphorus are described as follows.

3. Measurement of dissolved inorganic phosphorus(DIP). All phosphorus elements in this experiment were determined by Mo-Sb colorimetric method. The composition of the chromogenic solution was: 0.05% $SbK(C_4H_4O_6)_2$, 1% $(NH_4)_2MoO_4$, 1.5% Ascorbic Acid and 28.15% concentrated sulfuric acid. All reagents, except sulfuric acid, reached a purity of 99.8%. Samples were diluted before measurements, then mixed with a certain volume ratio of chromogenic solution and measured their OD_{700} using the spectrophotometry method.
The samples used in the measurements that do not involve microbial phosphorus are the centrifugal supernatant of the original liquid sample, including the measurement of DIP, DOP, and SMBP. In the process of these measurements, we set a water control and a supernatant control to eliminate the great interference caused by the color of the liquid sample itself and the chromogenic solution. The chromogenic agent was added to the water control and no chromogenic agent was added to the supernatant control. The sample, together with its corresponding supernatant control and water control was measured for

their OD_{700} using a microplate reader, and the value of OD_{700} was labeled as A_S, A_{SC} and A_{WC} respectively. We believe that the value of A_S minus A_{SC} and A_{WC} can be considered as the true absorbance of molybdenum-phosphorus hetero-poly blue produced by the reaction of DIP with Mo-Sb colorimetric chromogenic agent, and the concentration of DIP of the sample, C_{DIP}, can be calculated through the standard curve. This method was also used to eliminate interference in the following phosphorus measurements of liquid samples.

4. Measurement of dissolved organic phosphorus(DOP). For a centrifugal supernatant sample whose C_{DIP} has been measured, we measured the concentration of DOP by the following method. Firstly, 0.5 ml supernatant sample and 0.1 ml $K_2S_2O_8$ solution with a concentration of 50 mg/mL were added into a 10ml tube, then digested at 121℃ for 30 minutes, and diluted to 6 ml scale for chromogenic process and measurement of OD_{700} value. Control groups were also treated using the same method. The phosphorus concentration of the digested solution, labeled as C_1, was calculated according to the standard curve. The concentration of the total dissolved phosphorus of the sample, or C_{TDP}, can be calculated by the following formula:

$$(2)\ C_{TDP} = \frac{C_1 \times 6ml}{0.5ml + 0.1ml}$$

and the concentration of dissolved organic phosphorus, or the C_{DOP}, can be calculated by the following formula:

$$(3)\ C_{DOP} = C_{TDP} - C_{DIP}$$

5. Measurement of suspended microbial biomass phosphorus(SMBP). For a centrifugal supernatant sample whose C_{TDP} has been measured (here we ignore the minimal volume change caused by bacterial precipitation before and after centrifugation of the liquid sample, and the C_{TDP} of the liquid sample is equivalent to that of the centrifugal supernatant), the phosphorus content in the bacteria in the sample was determined by the following method. Take 0.4 ml bacterial solution into a 10 ml test tube, and add 0.8 ml NaOH solution with a concentration of 8 g/L. Shake the test tube gently to fully decompose the bacterial body until the liquid is clarified. Then, 0.6 ml of NaAC buffer with pH=4.8 was added for neutralization, and the concentration of total phosphorus in the liquid sample, or C_{TPLS}, was measured according to the method in Laboratory Measurement 3. Then, the concentration of the suspended microbial biomass phosphorus, or C_{SMBP}, was calculated by the following formula:

$$(4)\ C_{SMBP} = C_{TPLS} - C_{TDP}$$

The concentration of phosphorus measured by liquid or centrifugal supernatant samples was normalized into the mass ratio(ω) of the regolith simulant according to the mass ratio of the liquid phase and solid phase in the culture system(around 2). Only the mass ratio of all phosphorus forms was used in the analysis and plotting.

6. Measurement of the water content of solid samples. We first weighed the mass of the empty centrifuge tube, which was labeled as m_{tube}; Secondly, the solid sample was

loaded and weighed again. The total mass of the solid sample and the centrifugal tube was labeled as m_{wet}. The solid sample was dried slowly at 70℃ to constant weight, and weighed again, which was labeled as m_{dry}. The water content of the solid sample was calculated according to the following formula:

$$(5)\ \omega_{water} = 1 - \frac{m_{dry} - m_{tube}}{m_{wet} - m_{tube}} \times 100\%$$

7. Measurement of adsorbed phosphorus. According to the definition of absorbed phosphorus, which includes the phosphorus that is adsorbed with mineral particles but not dissolved in solution when the transformation between various forms of phosphorus elements reaches equilibrium at a certain time of culture, we used the Olsen method [1] described as follows: 0.4 g completely dried solid sample was loaded in 10 ml test tube, and 8 ml $NaHCO_3$ solution with a concentration of 0.5 mol/L was added, and the sample was shaken at 200 rpm, 25℃ for 30 minutes precisely. The extract solution was immediately filtered by non-phosphorus filter paper, and the Mo-Sb colorimetric method was used for colorimetric measurement of phosphorus concentration, or labeled as C2. The absorbed phosphorus of the dry solid sample was calculated by the following formula(in mass ratio):

$$(6)\ \omega_{AP} = \frac{C_2 \times 8ml}{0.4g} - \frac{\omega_{water}}{1 - \omega_{water}} \times C_{TPLS}$$

By using this method, we eliminated the influence of the phosphorus content in the liquid phase of solid samples.

8. Measurement of attached microbial biomass phosphorus(AMBP). We used chloroform fumigation to release phosphorus from the body of microorganisms. The specific operation was as follows: Firstly we weighed 0.4 g of dried solid sample in a 10 ml test tube and put it into a vacuum dryer together with chloroform. Then vacuumed and boiled the chloroform. After 24 hours of off-light treatment, the samples were repeatedly vacuumed to fully volatilize the chloroform from the regolith, until there was no chloroform odor. Then, the samples were extracted according to Laboratory measurement 6. The extract solution was later digested as Laboratory measurement 3. The total phosphorus concentration in the extract solution, labeled as C_{TPES}, was obtained by Mo-Sb colorimetric method. The mass ratio of AMBP can be calculated according to the following formula:

$$(7)\ \omega_{AMBP} = \frac{C_{TPES} \times 8ml}{0.4g} - \frac{\omega_{water}}{1 - \omega_{water}} \times C_{TPLS} - \omega_{AP}$$

9. Measurement of soluble salt content. Firstly, we weighed a 2 ml centrifugal tube on the analytical balance and record it as m_{tube}. Then, 1ml of the digested and diluted solution in the Laboratory measurement 3 was added and dried at 95℃ to prevent spatter loss during boiling. After drying, 30% H_2O_2 solution was added by drips until the color of the precipitate is white. Then dried the H_2O_2 solution to a constant weight of the centrifugal tube, and weighed as m'_{tube}. Considering that $K_2S_2O_8$ is converted into K_2SO_4 after digestion, the soluble salt concentration was calculated by the following formula:

$$(8)\ C_{salt} = \frac{(m'_{tube} - m_{tube}) \times - C_{K_2S_2O_8} \times 0.1mL \times M_{K_2SO_4}/M_{K_2S_2O_8}}{0.5mL}$$

10. Measurement of the diameter of plants at 24 DAS and Length of seedlings at 6 DAS. After 6 or 24 days of growth, the whole plant, including the root, of *Nicotiana benthamiana* was carefully removed from the substrate. The samples were arranged on a dark flat background, and a hand-held camera was used to photograph directly above the plants. In order to keep the plants close to the background plate, some of the cotyledon of the plants were removed and double-sided tape was used during imaging. The diameter of rosette leaves and the length of seedlings were measured by ImageJ. The horizontal projection distance of the longest leaf pairs was taken as the diameter of the rosette leaf. The length of the curve from the base of the cotyledon to the tip of the root was measured as the length of the seedling, and the length of the curve was measured using at least six anchor points.

11. Measurement of fresh weight of *Nicotiana benthamiana* plants. The plant samples were immediately transferred to a 2 ml centrifuge tube that had been marked, after they were photographed. The centrifuge tube containing a corresponding plant sample was weighed on an electronic analytical balance (accuracy 0.0001g) as m''_{tube}. The empty tube was weighed as m_{tube} earlier. The fresh weight of a plant sample was calculated by the following formula:

$$(9)\ m_{FW} = m''_{tube} - m_{tube}$$

12. Measurement of the chlorophyll content of leaves. After weighing, the uppermost fully unfolded leaf of each samples were picked and weighed again. All leaf samples from the same replicate(2-4 leaves per replicate) were transferred to a 2 ml centrifuge tube that had been marked. The total weight of the leaves were recorded as M_{leaves}. The chlorophyll content of the leaves was determined by thermal ethanol method, which is described as follows. V ml of anhydrous ethanol was add to the centrifuge tube, completely submerging the samples. The tubes were then heated to 80℃, and kept for 1 hour. The tubes were turned periodically during heating to ensure that the chlorophyll is fully extracted. The absorbance of the extract solution at 649 nm and 665 nm was subsequently determined on a spectrophotometer using anhydrous ethanol as a blank control. The chlorophyll content of leaves were calculated according to the empirical equation from Wintermans and De Mots [2]:

$$(10)\ C_{chlorophyll} = \frac{(OD_{649} \times 20.04 + OD_{665} \times 6.10) \times V}{M_{leaves}}$$

The chlorophyll content is expressed as milligrams of chlorophyll per gram of fresh weight (mg/g).

13. Measurement of soluble salt content. An additional soil sample was taken for each treatment at the beginning of culture, sowing, and 24 DAS, for soluble salt content measurement. The samples were firstly weighed as M_{soil}, then extracted at 25℃ with 1 ml

of ddH_2O for 0.5 hour. 0.5 ml of the extract solution was then added to a 10 ml centrifuge tube weighed as M_{tube}, and then dried in a vacuum drying oven at low temperature, to prevent thermal decomposition. After drying, the tube was weighed and recorded as M_{salt}. The soluble salt content was calculated by the following fomula:

$$(11)\ C_{salt} = \frac{(M_{salt} - M_{tube}) \times \frac{1}{0.5} ml}{M_{soil}}$$

It should be noted: in order to ensure that the sampling procedure does not cause too much interference to the plant growth, we cannot obtain enough samples during the growth stage of the plant to determine the salt content.

14. Measurement of soil extract: pH and OD_{600}. The sample used for pH, OD600 and available phosphorus content measurement were divided into two parts. The smaller part was used to measure its water content, according to the method described in section 5. The water content of the small part of sample was considered to be the same as that of the larger sample.

The larger part was used for further measurement. It was weighed as M_{soilFW}, and its dry weight M_{dry} was calculated by the following fomula:

$$(12)\ M_{dry} = M_{soilFW} \times (1 - \omega_{water})$$

The purpose of determining the water content of the larger samples by calculation, rather than by drying, was to ensure the survival of microbes, and the chemical properties were as consistent as they were when cultivating plants.

The larger part of the soil sample was then extracted at 25℃ for 0.5 hour, with ddH_2O twice of its dry weight. Then 100 μl of the extract solution was transferred to a 96-well plate for OD_{600} measurement. The measurement was done on microplate reader(Multiskan FC, Thermo Fisher Scientific). Then, the 100 μl extract solution was added back to the sample, instead of discarding. The porpose of this step was to prevent the interference on further measurement. The pH of the sample was later measured using a electronic pH meter.

15. Measurement of available phosphorus content of the soil. The measurement of phosphorus was conducted right after the measurement of pH. $NaHCO_3$ solution with a concentration of 0.625 mol/L and a volume eight times the dry weight value of the sample was added to the sample, so that the final ratio of water and soil was 10:1, and the final concentration of $NaHCO_3$ was 0.5 mol/L. The sample was then extracted at 25℃ for 0.5 hour, with a shaking speed of 200 rpm. The following steps were as described in section 6.

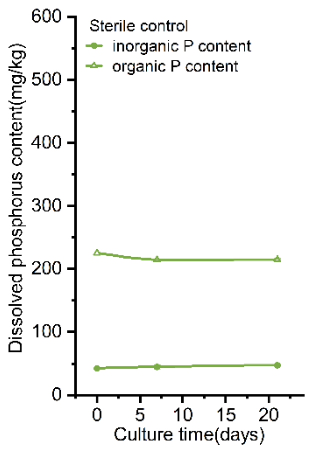

Supplementary figure 1 The dynamics of the dissolved inorganic P and dissolved organic P content in the Sterile control. In the control group of the phosphorus measurement, the medium mixed with simulant was not inoculated with PSBs. All error bars represent the Standard deviation. The phosphorus content in the control group should be understood as the phosphorus contained in the glucose broth medium.

Supplementary Table 1 The numbers of available replicates in all treatments at different period of experiment.

Experiment	Treatment	Numbers of available replicates						
		0 DAI	2 DAI	4 DAI	7 DAI	10 DAI	15 DAI	21 DAI
PSB culture experiment	*B. mucilaginosus*	4	3	2	2	2	2	2
	B. megaterium	4	4	4	4	4	4	4
	B. subtilis	4	3	3	3	3	3	3
	B. licheniformis	4	4	3	3	3	3	3
	P. fluorescens	4	4	4	4	4	4	4

Experiment	Treatment	Numbers of available replicates							
		-18 DAS	-12 DAS	-6 DAS	Sowing	6 DAS	12 DAS	18 DAS	24 DAS
Nicotiana benthamiana cultivation experiment	Blank control	12	12	12	12	12	12	12	12
	Sterilized control	12	12	12	12	12	12	12	12
	Not pre-cultured	12	12	12	12	12	12	12	12
	Pre-cultured for 6 days	12	12	12	12	12	12	12	12
	Pre-cultured for 12 days	12	12	12	12	12	12	12	12
	Pre-cultured for 18 days	12	12	12	12	12	12	12	12

Supplementary Reference

[1]Olsen, S. R. et al. Estimation of available phosphorus in soil by extraction with sodium bicarbonate. USDA Circular No. 939, US Government Printing Office, Washington DC(1951).

[2]Wintermans, J.E.G. and De Mots, A. Spectrophotometric Characteristics of Chlorophyll a and b and Their Phaeophytins in Ethanol. *Biochimica et Biophysica Acta*, **109**, 448-453 (1965).
