# Supplementary material for: Phosphorus-solubilizing bacteria improve the growth of Nicotiana benthamiana on lunar regolith simulant by dissociating insoluble inorganic phosphorus
Source: Commun Biol. 2023 Nov 9;6:1039. doi: 10.1038/s42003-023-05391-z (PMC10636133; doi:10.1038/s42003-023-05391-z)
Supplement: Supplementary file 3 — Description of Supplementary Materials [file 42003_2023_5391_MOESM3_ESM.docx]

**Description of Additional Supplementary Files**

**File name:** Supplementary Data

**Description:**The source data behind the graphs and tables in the manuscript
