# Supplementary material for: Phosphorus-solubilizing bacteria improve the growth of Nicotiana benthamiana on lunar regolith simulant by dissociating insoluble inorganic phosphorus
Source: Commun Biol. 2023 Nov 9;6:1039. doi: 10.1038/s42003-023-05391-z (PMC10636133; doi:10.1038/s42003-023-05391-z)
Supplement: Supplementary file 5 — Reporting summary [file 42003_2023_5391_MOESM5_ESM.pdf]

nature portfolio

Corresponding author(s): Zhencai Sun
Last updated by author(s): Sep 22, 2023

Reporting Summary

Nature Portfolio wishes to improve the reproducibility of the work that we publish. This form provides structure for consistency and transparency in reporting. For further information on Nature Portfolio policies, see our Editorial Policies and the Editorial Policy Checklist.

Statistics

For all statistical analyses, confirm that the following items are present in the figure legend, table legend, main text, or Methods section.

n/a	Confirmed	
☐	☒	The exact sample size (*n*) for each experimental group/condition, given as a discrete number and unit of measurement
☐	☒	A statement on whether measurements were taken from distinct samples or whether the same sample was measured repeatedly
☐	☒	The statistical test(s) used AND whether they are one- or two-sided<br>*Only common tests should be described solely by name; describe more complex techniques in the Methods section.*
☐	☒	A description of all covariates tested
☐	☒	A description of any assumptions or corrections, such as tests of normality and adjustment for multiple comparisons
☐	☒	A full description of the statistical parameters including central tendency (e.g. means) or other basic estimates (e.g. regression coefficient) AND variation (e.g. standard deviation) or associated estimates of uncertainty (e.g. confidence intervals)
☐	☒	For null hypothesis testing, the test statistic (e.g. F, t, r) with confidence intervals, effect sizes, degrees of freedom and P value noted<br>*Give P values as exact values whenever suitable.*
☐	☒	For Bayesian analysis, information on the choice of priors and Markov chain Monte Carlo settings
☐	☒	For hierarchical and complex designs, identification of the appropriate level for tests and full reporting of outcomes
☐	☒	Estimates of effect sizes (e.g. Cohen's *d*, Pearson's *r*), indicating how they were calculated

Our web collection on statistics for biologists contains articles on many of the points above.

Software and code

Policy information about availability of computer code

Data collection	The software that we used to collect data from micro-plate reader was skanit, version 6.1.1.
Data analysis	The softwares that we used to analysis data were Microsoft Excel, version 2021, Origin lab, version 2023, SPSS, version 29.0.0, and ImageJ, version Fiji.

For manuscripts utilizing custom algorithms or software that are central to the research but not yet described in published literature, software must be made available to editors and reviewers. We strongly encourage code deposition in a community repository (e.g. GitHub). See the Nature Portfolio guidelines for submitting code & software for further information.

Data

Policy information about availability of data

All manuscripts must include a data availability statement. This statement should provide the following information, where applicable:

- Accession codes, unique identifiers, or web links for publicly available datasets
- A description of any restrictions on data availability
- For clinical datasets or third party data, please ensure that the statement adheres to our policy

Data supporting the findings of this work are available within the paper and in the Supplementary files. The data sets generated and analyzed for this study are available from the corresponding author upon request.

Human research participants

Policy information about studies involving human research participants and Sex and Gender in Research.

Reporting on sex and gender	No information about sex or gender was collected.
Population characteristics	No information about population characteristics was collected.
Recruitment	No participant was recruited.
Ethics oversight	No ethic approval was need for our study.

Note that full information on the approval of the study protocol must also be provided in the manuscript.

Field-specific reporting

Please select the one below that is the best fit for your research. If you are not sure, read the appropriate sections before making your selection.

☒ Life sciences ☐ Behavioural & social sciences ☐ Ecological, evolutionary & environmental sciences

For a reference copy of the document with all sections, see nature.com/documents/nr-reporting-summary-flat.pdf

Life sciences study design

All studies must disclose on these points even when the disclosure is negative.

Sample size	For measurement of elemental composition of regolith simulant: n=3. For Standard test for the ability of microbes to dissolve insoluble inorganic phosphorus: n=3. For shaking flask experiment: controls without simulant, n=1; controls without PSBs, n=4; other treatments, n=4. For cultivation experiment, n=12 for analysis of soil samples; n=12,35,21,21, 36,43,32 for the group of "Horticultural soil","Blank control","Sterilized control","Not-pre-cultured","Pre-cultured for 6 days","Pre-cultured for 12 days" and "Pre-cultured for 18 days" respectively.
Data exclusions	For the shaking flask experiment, the replicate #D of B. mucilaginosus was excluded since 7 DAI; the replicate #D of B. subtilis was excluded since 2 DAI; the replicate #D of B. licheniformis was excluded since 4 DAI. All these exclusions were made due to the infection of environmental microbes. For cultivation experiment, the replicate #C1 was excluded, as there was no living plants at 24 DAS, unable to conduct related analysis.
Replication	We have conducted two rounds of cultivation experiment, with a smaller one of 5 replicates and a larger one of 12 replicates. We have observed similar results from both experiments that the PSBs treatment could promote the growth of plants in the lunar regolith simulant, which gave us the confidence that the findings of our manuscript was able to be replicated. The experiment presented in our manuscript is the one with a larger scale.
Randomization	when culturing PSBs or cultivating plants, the flasks or 24-well plates were placed randomly in the incubating box and adjusted periodically, so that the changes in environmental conditions (temperature, light, etc.) have a uniform effect on all treatment groups and can therefore be excluded in statistical analysis
Blinding	The study did not involve any human participants, therefore blinding is not relevant.

Reporting for specific materials, systems and methods

We require information from authors about some types of materials, experimental systems and methods used in many studies. Here, indicate whether each material, system or method listed is relevant to your study. If you are not sure if a list item applies to your research, read the appropriate section before selecting a response.

Materials & experimental systems

n/a	Involved in the study	
☒	☐	Antibodies
☒	☐	Eukaryotic cell lines
☒	☐	Palaeontology and archaeology
☒	☐	Animals and other organisms
☒	☐	Clinical data
☒	☐	Dual use research of concern

Methods

n/a	Involved in the study	
☒	☐	ChIP-seq
☒	☐	Flow cytometry
☒	☐	MRI-based neuroimaging
